# CTCF-silenced miR-137 contributes to EMT and radioresistance in esophageal squamous cell carcinoma

**DOI:** 10.1186/s12935-020-01740-8

**Published:** 2021-03-08

**Authors:** Shuwen Xu, Xiaofeng Li, Longfei Li, Yufeng Wang, Chong Geng, Feng Guo, Tao Zhang, Aonan Du, Zhiwei Lu, Hua Hui, Qiang Wang

**Affiliations:** 1 Department of Head and Neck Surgery, Xuzhou Cancer Hospital, Xuzhou, 221005 Jiangsu China; 2 Department of Radiology, Xuzhou Cancer Hospital, Xuzhou, 221005 Jiangsu China; 3 Department of Cardiothoracic Surgery, Xuzhou Cancer Hospital, Xuzhou, 221005 Jiangsu China; 4 Department of Nuclear Medicine, Xuzhou Cancer Hospital, Xuzhou, 221005 Jiangsu China; 5 Department of Radiation Oncology, Xuzhou Cancer Hospital, No. 131, Huancheng Road, Gulou District, Xuzhou, 221005 Jiangsu China

**Keywords:** ESCC, CTCF, miR-137, EZH2, PXN

## Abstract

**Background:**

Esophageal squamous cell carcinoma (ESCC) is one of the most malignant tumors in gastrointestinal system. MicroRNAs (miRNAs) have been reported to be implicated in cancer development. However, the role of miR-137 has not been fully revealed in ESCC.

**Methods:**

Quantitative real-time polymerase chain reaction (qRT-PCR) and western blot analyses were separately used to examine RNA level and protein level. 5-ethynyl-2′-deoxyuridine (EdU) assay, transwell assays and flow cytometry analyses were conducted to assess biological behaviors of ESCC cells. Additionally, the interaction between genes were analyzed via Chromatin Immunoprecipitation (ChIP) assay, RNA Binding Protein Immunoprecipitation (RIP) assay, RNA pull down assay and luciferase reporter assay.

**Results:**

MiR-137 was down-regulated in ESCC cells. Upregulation of miR-137 hindered ESCC cell proliferation, migration, invasion and epithelial mesenchymal transition (EMT). Besides, miR-137 enhanced the sensitivity of ESCC cells to irradiation. Moreover, CCCTC-binding factor (CTCF) inactivated miR-137 transcription in ESCC cells. Furthermore, we revealed enhancer of zeste 2 polycomb repressive complex 2 subunit (EZH2) and paxillin (PXN) as the downstream targets of miR-137. In turn, EZH2 was recruited by CTCF and induced methylation in miR-137 promoter.

**Conclusion:**

CTCF/Suz12/EZH2 complex-silenced miR-137 facilitates ESCC progression and radioresistance by targeting EZH2 and PXN.

## Background

Esophageal carcinoma (EC) occurs in esophageal epithelial tissue, is one of the commonest malignancies with leading causes of cancer-associated mortality globally [1]. The deaths cases of EC reach approximately 509,000 each year [2]. Esophageal adenocarcinoma (EA) and esophageal squamous cell carcinoma (ESCC) are two major subtypes of EC. ESCC remains the predominant subtype in China, which accounts for almost 90% of all EC cases [3]. Chemotherapy [4] and radiotherapy [5] has been applied in the treatment for ESCC patients. However, chemoresistance and radioresistance will occur in advanced stage. Therefore, exploring the molecular mechanism is necessary for improving the effectiveness of chemotherapy and radiotherapy.

Alteration of RNA expression is closely correlated with the progression of human diseases [6, 7]. Non coding RNAs (ncRNAs) have been reported in recent years due to their crucial role in human cancers [8]. MicroRNAs (miRNAs) are a class of short ncRNA molecules that engaged in diverse biological activities [9–12]. Numerous miRNAs have been reported to be dysregulated and is associated with the development of various cancers [13–22], including EC [23]. MiR-137 has been validated to be a tumor suppressor in several cancer types. For instance, miR-137 is lowly expressed in pancreatic cancer tissues and cell lines and suppresses the progression of pancreatic cancer [24]. Furthermore, miR-137 restrains endometrial cancer cell proliferation [25]. Downregulation of miR-137 in endometrial cancer potentially due to the methylation of miR-137 promoter [26]. However, the role miR-137 in ESCC progression has never been investigated.

Enhancer of zeste homolog 2 (EZH2) is an enzymatic subunit of the polycomb repressive complex 2 (PRC2) that plays crucial role in cancer development. EZH2 inhibition induces anti-myeloma impacts on multiple myeloma [27]. EZH2 can strengthen the resistance of gastric cancer to 5-FU [28]. Paxillin (PXN) has been found to be markedly abundant in cervical cancer tissues and is positively correlated with tumor stage and lymphatic metastasis [29]. To our knowledge, the interaction between miR-137 and EZH2 or PXN remains to be explored in ESCC.

This study was aimed to investigate the function of miR-137 in ESCC progression and unveiled its upstream or downstream molecular mechanism.

## Materials and methods

### Cell culture

Four human ESCC cell lines (Eca109, EC9706, KYSE150, KYSE450) and human normal esophageal epithelial cell line (Het-1A) were all procured from the ATCC (Manassas, VA, USA) and propagated at 37 °C in a humidified incubator of 5% CO_2_. Cell lines were cultured in RPMI-1640 medium (Gibco, Carlsbad, CA, USA) with 10% FBS (Gibco) and 1% Pen/Strep mixture.

### RNA extraction and quantitative real-time polymerase chain reaction (qRT-PCR)

Total RNA extraction from cell samples were achieved using Trizol method, then cDNA template was synthesized using complementary DNA (cDNA) reverse transcription kit (Applied Biosystems, Carlsbad, CA, USA). The Power SYBR® Green Master mix (Applied Biosystems) was used for qRT-PCR on StepOne™ Real-Time PCR System (Applied Biosystems). Glyceraldehyde-3-phosphate dehydrogenase (GAPDH) or U6 snRNA (U6) was regarded as the internal reference. The 2^−ΔΔCt^ method was applied to calculate relative gene expression.

### Transfection

MiR-137 mimics and negative control (NC) mimics were designed by Genepharma Company (Shanghai, China) to overexpress miR-137. The pcDNA3.1 vectors containing whole sequence of CCCTC-binding factor (CTCF), SUZ12 polycomb repressive complex 2 subunit (Suz12), EZH2 and paxillin (PXN) were synthesized by Genepharma and named as pcDNA3.1/CTCF, pcDNA3.1/Suz12, pcDNA3.1/EZH2 and pcDNA3.1/PXN. The empty pcDNA3.1 vector was used as NC. Transfections were accomplished using Lipofectamine2000 (Invitrogen, Carlsbad, CA, USA). Forty-eight hours later, Cells were reaped for analysis.

### 5-ethynyl-2′-deoxyuridine (EdU) assay

Cells were seeded to 96-well plates which were added with the EdU medium (Ribobio, Guangzhou, China) in PBS, then fixed with 4% paraformaldehyde for fixing. Cells were cultured with DAPI solution for 30 min and observed under a fluorescence microscope (Olympus, Tokyo, Japan).

### Transwell assay

Cells in serum-free medium were placed on the top of transwell chamber (BD Biosciences, Franklin Lakes, NJ, USA) pre-coated with or without Matrigel for invasion or migration assay. The conditioned medium served as the supplements of lower chamber. After incubated for 24 h, cells on the bottom were fixed and dyed with 0.5% crystal violet. Five fields in each well were counted randomly.

### Western blot

Total protein extracts were acquired using RIPA lysis buffer, then dissolved in 12% SDS-PAGE gel and transferred to PVDF membranes. After blocking in 5% skim milk for 1 h, the membranes were incubated with the primary antibody against GAPDH (the internal control), ZO-1, E-cadherin, N-cadherin, Vimentin, CTCF, Suz12, EZH2, PXN, and then were incubated with the secondary antibodies conjugated to HRP. All antibodies were purchased from Abcam (Cambridge, MA, USA). At last, protein blots were exposed to the enhanced chemiluminescence (ECL) fluorescence test kit (Amersham, Arlington Heights, IL) (Additional file 1: Fig. S1).

### Survival fraction assay

Transfected ESCC cells in 6-well plates were processed with the irradiation at doses of 0, 2, 4, 6, 8 Gy, then cultured in medium at 37 °C for colony formation. Fourteen days later, cells were fixed and stained with 0.1% crystal violet. Survival clones with > 50 cells were counted manually under Olympus microscope.

### Flow cytometer of apoptosis

Cell apoptosis was analyzed by Annexin-V fluorescein isothiocyanate (FITC)/propidium iodide (PI) dual-staining kit as per the instruction. The apoptotic cells were detected by use of FACSC alibur flow cytometer (BD Biosciences) with CellQuest software (BD Biosciences).

### Chromatin immunoprecipitation (ChIP)

ESCC cells were fixed in 4% paraformaldehyde for 10 min to form cross-linking of DNA and protein. After ultrasonic, antibodies against CTCF, Suz12, EZH2, H3K27Me3 and control IgG antibody (Millipore, Billerica, MA, USA) were prepared for the immunoprecipitation. The precipitated DNA was recovered by magnetic beads for qRT-PCR analysis.

### Luciferase reporter assay

MiR-137 promoter covering the CTCF binding sites was amplified and sub-cloned into pGL3-basic vector (Promega, Madison, WI, USA), and then co-transfected into HEK-293 T cells (ATCC) with pcDNA3.1/CTCF or empty pcDNA3.1 vector. Besides, the EZH2 or PXN fragments containing wild-type and mutated miR-137 binding sites were applied for the establishment of EZH2-WT/Mut and PXN-WT/Mut using pmirGLO vector (Promega), then co-transfected with miR-137 mimics or miR-NC in HEK-293 T cells. Fluorescence intensity changes were tested by Dual-Luciferase Reporter Assay System (Promega).

### RNA immunoprecipitation (RIP)

Cells in complete RIP lysis buffer were collected and incubated with the beads-bound anti-Ago2 antibody or anti-IgG antibody for 2 h in RIP buffer. After immunoprecipitation, the RNAs-bound to protein were isolated for qRT-PCR.

### RNA pull down assay

The wild-type and mutant interacting sites of EZH2 or PXN in miR-137 sequences were synthesized and biotinylated severally into Bio-miR-137-WT/Mut, followed by mixing with the cellular protein extracts. Beads were added to collect precipitated mixtures, then analyzed by qRT-PCR.

### Immunofluorescence (IF) staining

ESCC cells samples on coverslips were treated with 4% paraformaldehyde for permeabilizing, then blocked in 5% BSA. The primary antibodies against CTCF, Suz12, EZH2 (Abcam) were diluted and used as instructed by supplier. After washing in PBS, samples were cultured with secondary antibodies. Images were captured using a fluorescence microscope.

### Statistical analyses

All data from independent bio-triplicates were shown as the mean ± standard deviation (SD) and analyzed by Prism 6.0 software. Analysis of group difference was achieved by Student’s *t*-test (two-tailed) or one-way analysis of variance, with *p* < 0.05 as statistical significance.

## Results

### MiR-137 is expressed at a low level in ESCC cells and suppresses cell proliferation, migration and radioresistance

**Fig. 1:**
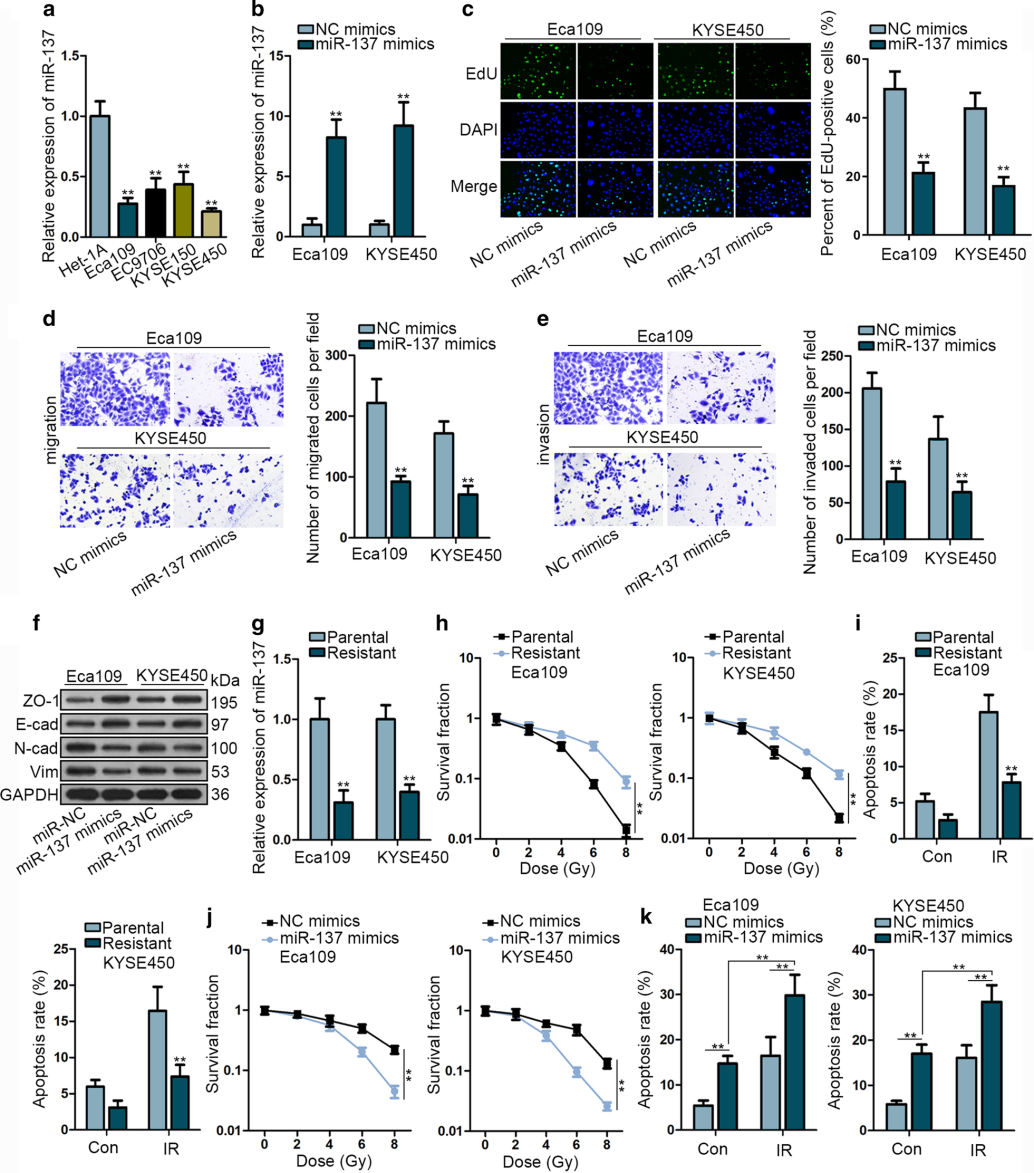
MiR-137 is expressed at a low level in ESCC cells and suppresses cell proliferation, migration and radioresistance. **a** The expression of miR-137 was evaluated by qRT-PCR assay in ESCC cell lines (Eca109, EC9706, KYSE150, KYSE450) and normal esophageal epithelial cell line (Het-1A). **b** The overexpression efficiency of miR-137 mimics was assessed by qRT-PCR. **c** The proliferation ability was testified by EdU assay in Eca109 and KYSE450 cells transfected with miR-137 mimics or NC mimics. **d**, **e** Transwell assays were performed to evaluate the migratory and invasive capacities after transfected with miR-137 mimics. **f** Western blot assays were conducted to measure the level of EMT-related proteins in two ESCC cells with miR-137 upregulation. **g** The expression of miR-137 was detected by qRT-PCR assay in parental Eca109 and KYSE450 cells and radio-resistant cells. **h** Clonogenic survival assay showed that the surviving fraction of radio-resistant cells and parental cells. **i** Flow cytometry analysis was performed to evaluate cell apoptosis rate in parental and radio-resistant cells. **j** Clonogenic survival assay was performed to testify radio sensitivity after transfected with miR-137 mimics. **k** Flow cytometry analysis was performed to evaluate cell apoptosis rate after transfected with miR-137 mimics. **P < 0.01

Firstly, we utilized qRT-PCR to explore miR-137 expression in ESCC cells. We observed that the expression of miR-137 was markedly lower in ESCC cell lines compared with that in the normal cell line Het-1A (Fig. 1a). Since miR-137 was expressed at lowest level in Eca109 and KYSE450, we chose these two cell lines for substantial experiments. For gain-of-function assay, miR-137 was overexpressed through transfecting with miR-137 mimics (Fig. 1b). EdU assay manifested that miR-137 mimics significantly decreased the percent of EdU positive cells (Fig. 1c). Transwell assay revealed that upregulation of miR-137 visibly impeded the migratory and invasive abilities of two ESCC cells (Fig. 1d, e). Additionally, western blot was conducted to measure the protein level of EMT-related proteins. The results indicated that ectopic expression of miR-137 could reverse EMT process (Fig. 1f).

To explore whether miR-137 exerted effects on radioresistance in ESCC cells, we performed qRT-PCR to examine its expression in parental cell line and radio-resistant cell lines. We identified the relative low level of miR-137 in radio-resistant Eca109 and KYSE450 cells (Fig. 1g). Clonogenic survival assay manifested that the surviving fraction was notably increased in radio-resistant Eca109 and KYSE450 cells compared with that of untreated parental ones (Fig. 1h). Flow cytometry analysis showed that the apoptotic rate was lower in radio-resistant cells than that in parental ones with or without irradiation (Fig. 1i). After steadily irradiating with a dose of x-ray radiation, miR-137 mimics significantly reduced the surviving fraction compared with NC mimics group, indicating that miR-137 could suppress radioresistance of ESCC cells (Fig. 1j). Additionally, miR-137 mimics markedly increased the ratio of apoptosis of ESCC cells treated with or without irradiation. (Fig. 1k). This phenomenon revealed that miR-137 could inhibit the resistance of ESCC cells to irradiation.

### CTCF represses the transcription of miR-137

**Fig. 2:**
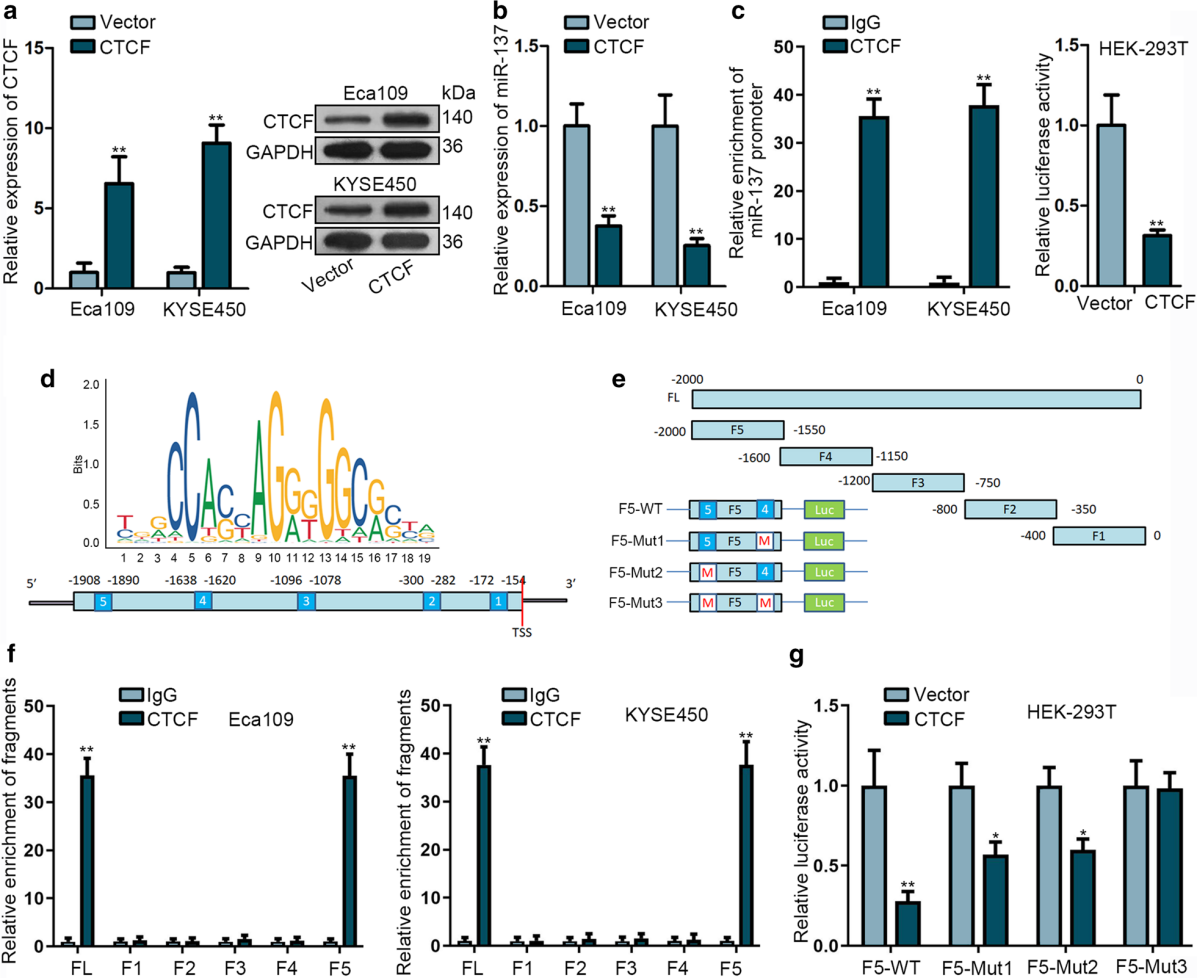
CTCF represses the transcription of miR-137. **a** qRT-PCR and western blot assays were performed to determine the level of CTCF mRNA and protein. **b** The expression of miR-137 was examined after CTCF overexpression. **c** ChIP assays were conducted to investigate the affinity of CTCF in miR-137 promoter (left). Luciferase reporter was conducted to assess the effect of CTCF on the transcriptional activity of miR-137 in HEK-293 T cell (right). **d** Potential binding site between CTCF and miR-137 promoter region. **e** The promoter region of miR-137 was divided into five segments and the sequence of F5 was mutated. **f** ChIP assay was performed to explore the specific fragment in miR-137 promoter responsible for the binding with CTCF. **g** Luciferase reporter was conducted to determine which sequence in F5 fragment could bind to CTCF. *P < 0.05, ***P < 0.01

Transcription factor could regulate the expression of miRNAs through activating or repressing transcription. We detected that CTCF might be a transcription factor of miR-137 by utilizing JASPAR (http://jaspardev.genereg.net/). We up-regulated the expression of CTCF in Eca109 and KYSE450 cells firstly (Fig. 2a, b). ChIP assay showed that CTCF could bind to miR-137 promoter (Fig. 2c). Then, we obtained DNA motif of CTCF and its binding sites in miR-137 promoter from JASPAR (Fig. 2d). We divided the promoter region into five fragments for further experiments (Fig. 2e). Later, ChIP assay was conducted and the result revealed that F5 was responsible for the affinity (Fig. 2f). To elucidate which binding sites in F5 functions, we constructed F5-WT, F5-Mut1, F5-Mut2 and F5-Mut3 with corresponding sequence mutation. The promoter activity of F5-WT and F5-Mut3 were much impaired by CTCF, while this effect on promoter activity was partially abolished by F5-Mut1 or F5-Mut (Fig. 2g). Overall, these data implied that CTCF repressed the transcription of miR-137 in ESCC via binding to miR-137 promoter region at both 1638–1620 and − 1908–1890 site in the upstream of transcription start site (TSS).

### EZH2 and PXN are downstream targets of miR-137

**Fig. 3:**
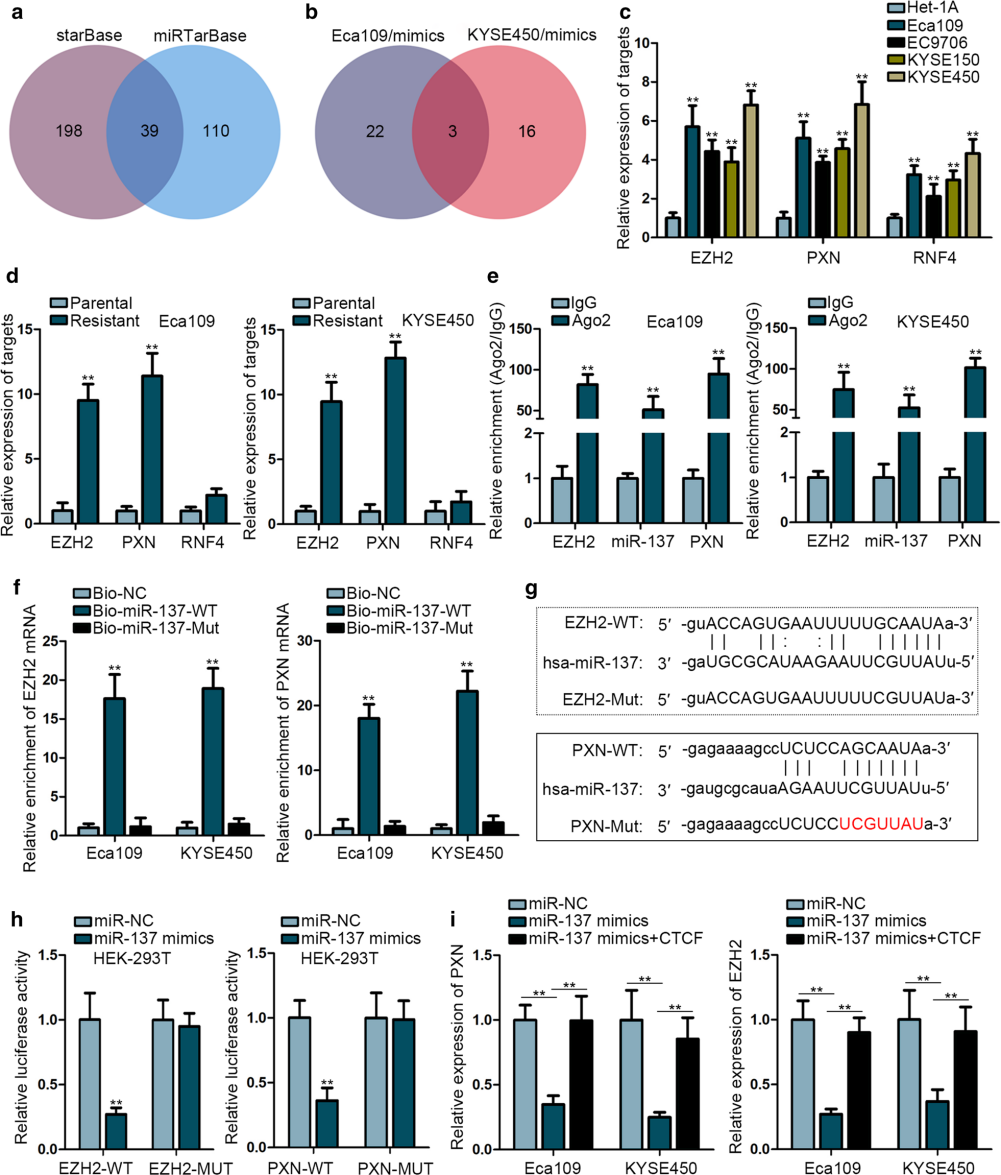
EZH2 and PXN are two downstream targets of miR-137.** a** The putative targets of miR-137 were predicted by utilizing starBase and miRTarBase bioinformatics tools. **b** Results of qRT-PCR analysis showed the mRNAs that were downregulate by the miR-137 upregulation in Eca109 and KYSE450 cells. **c** qRT-PCR was performed to detect the expressions of target genes in four ESCC cell lines and the normal cell line. **d** qRT-PCR was performed to evaluate the expressions of EZH2, PXN and RNF4 in parental and radio-resistant cells. **e** RIP assay manifested that miR-137, EZH2 and PXN were significantly enriched in Ago2 group. **f** RNA pull down showed that Bio-miR-137-WT could pull down EZH2 and PXN. **g** The binding sites between miR-137 and EZH2 or PXN. **h** Luciferase reporter assay was performed to validate the interaction between miR-137 and EZH2 or PXN. **i** The expression of PXN or EZH2 was measured by qRT-PCR in cells transfected with NC mimics, miR-137 mimics or miR-137 mimics + CTCF. **P < 0.01

Extensive documents have elucidated that miRNAs could induce translational repression or degradation of target mRNAs, leading to alteration of biological functions. Utilizing bioinformatics analysis, we screened 39 potential downstream targets of miR-137 (Fig. 3a). Among which, only three mRNAs could be downregulated by the upregulation of miR-137 in both ESCC cells (Fig. 3b). Further qRT-PCR analyses showed that EZH2 and PXN were expressed higher in ESCC cell lines than that in normal cell line (Fig. 3c) and were expressed at high level in radio-resistant Eca109 and KYSE450 cells than that in parental cells (Fig. 3d). RIP assay demonstrated the enrichment of miR-137, EZH2 and PXN in the Ago2-bound complexes (Fig. 3e), indicating their coexistence in RNA induced silencing complex (RISC). Furthermore, RNA pull down assay manifested that both EZH2 and PXN were preferentially abundant by Bio-miR-137-WT (Fig. 3f). Next, we found putative binding site of miR-137 with EZH2 and PXN and mutated the binging sites, respectively (Fig. 3g). We noticed that the luciferase activity of EZH2-WT or PXN-WT was attenuated dramatically by miR-137 mimics (Fig. 3h). Finally, the mRNA levels of PXN and EZH2 was depleted by miR-137 mimics, but was recovered again by co-repression of CTCF (Fig. 3i). Together, these findings revealed that EZH2 and PXN are two downstream targets of miR-137.

### CTCF induces DNA methylation of miR-137 via recruiting PRC2 complex

**Fig. 4:**
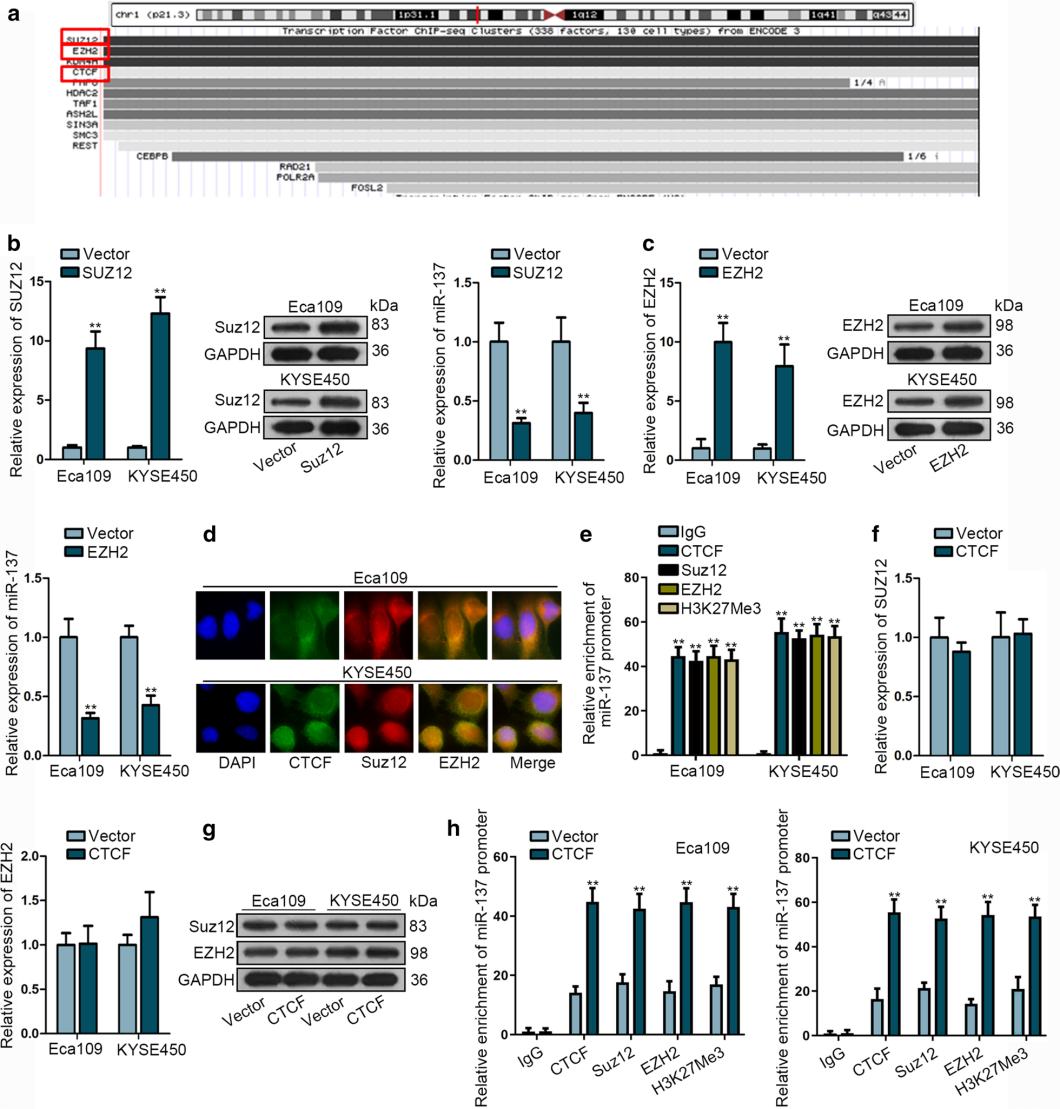
CTCF induces DNA methylation of miR-137 via recruiting PRC2 complex. **a** The potential transcription factor for miR-137 was predicted by UCSC tool. **b** qRT-PCR and western blot assays were performed to determine the level of Suz12 mRNA and protein as well as miR-137 expression after Suz12 overexpression. **c** qRT-PCR and western blot assays were performed to determine the level of EZH2 mRNA and protein as well as miR-137 expression after EZH2 overexpression. **d** Immunofluorescence assay was performed to determine the co-localization of CTCF, Suz12 and EZH2. **e** ChIP assay was conducted to test the affinity of CTCF, SUZ12, EZH2 and H3K27Me3 to miR-137 promoter. **f**, **g** qRT-PCR and western blot assays were performed to determine the level of Suz12 and EZH2 mRNA and protein in CTCF-abundant cells. **h** ChIP data indicated that Suz12 and EZH2 were recruited by CTCF to enhance the H3K27Me3 level of miR-137. **P < 0.01

Using UCSC (http://genome.ucsc.edu/), we focused on three potential transcription factors of miR-137, namely, Suz12, EZH2 and CTCF (Fig. 4a). It has been reported that CTCF can interact with suppressor of zeste 12 homologue (Suz12) and induces DNA methylation of downstream targets via polycomb repressive complex 2 (PRC2) complex [30]. Here, we conjectured that the depletion of miR-137 might be associated with the interaction between CTCF and PRC2 complex. We separately overexpressed Suz12 and EZH2 in Eca109 and KYSE450 cells and found that the level of miR-137 was decreased (Fig. 4b, c). To certify whether CTCF could interact with Suz12, we firstly examined the subcellular situation of CTCF, Suz12 and EZH2 and found that all of them were predominantly located in the nucleus of Eca109 and KYSE450 cells (Fig. 4d). Histone H3 lysine 4 trimethylation (H3K4me3) was closely related with transcriptional activity and open-chromatin structures, and served as a repressive marker of transcription [31]. ChIP assay showed that the enrichment of miR-137 promoter in the immunoprecipitation with CTCF, Suz12, EZH2 and H3K27Me3 antibodies (Fig. 4e). qRT-PCR and western blot analyses revealed that CTCF had no effects on the expression of Suz12 and EZH2 mRNA and protein (Fig. 4f, g). ChIP data revealed that the upregulation of CTCF enhanced the enrichment of miR-137 promoter in CTCF, Suz12, EZH2 group and increased H3K27Me3 level (Fig. 4h). These findings suggested that CTCF interacts with Suz12/EZH2 and induces DNA methylation of miR-137 promoter via recruiting PRC2 complex.

### MiR-137 inhibits ESCC cell proliferation, migration and radioresistance by targeting EZH2 and PXN

**Fig. 5:**
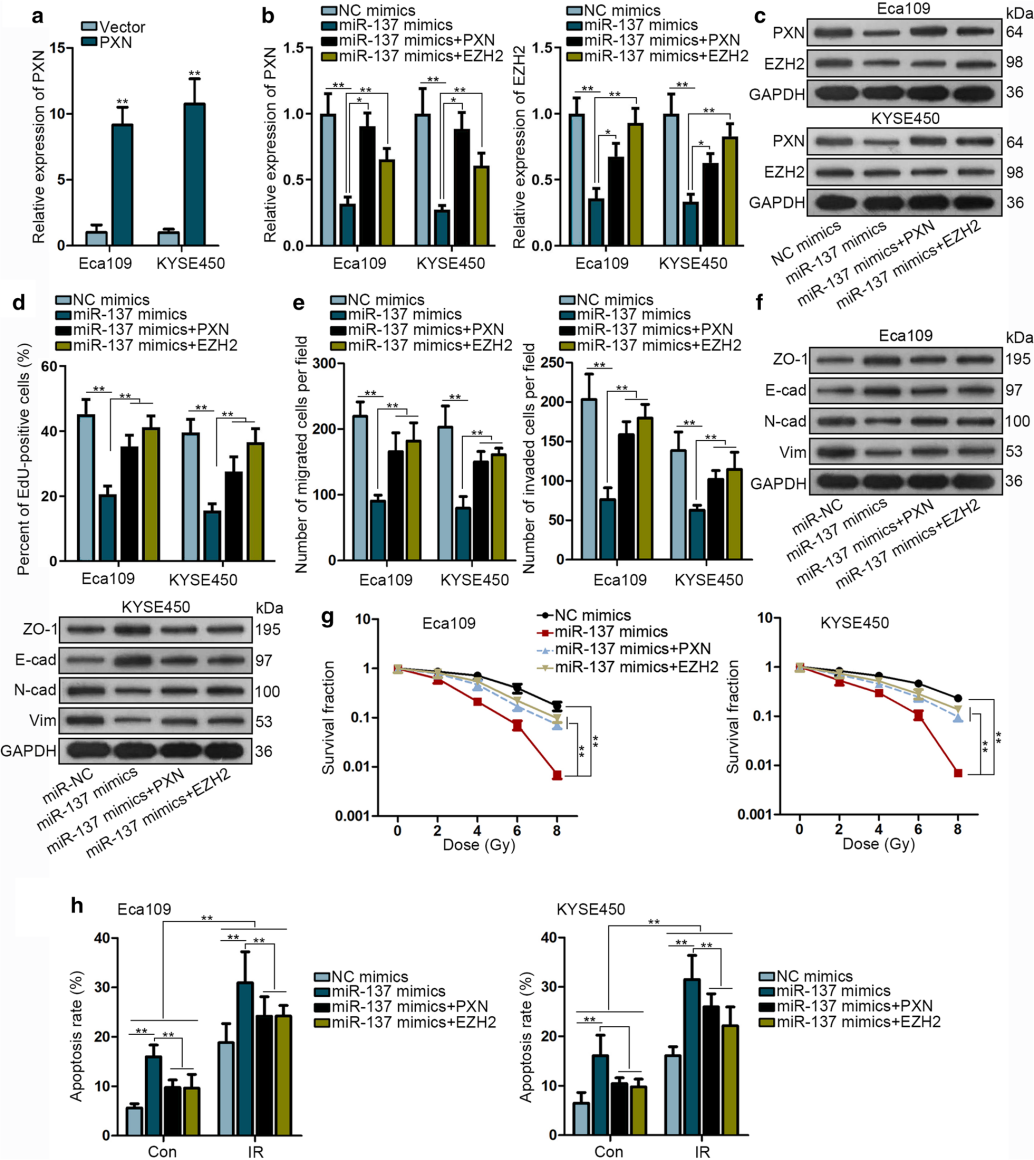
MiR-137 inhibits ESCC cell proliferation, migration and radioresistance by targeting EZH2 and PXN. **a** qRT-PCR analysis was utilized to determine the overexpression of PXN. **b**, **c** The level of PXN and EZH2 mRNA and protein were measured by qRT-PCR and western blot assays in indicated cells. **d** EdU assay was conducted to measure the effects of PXN or EZH2 on miR-137 mimics-mediated cell proliferation. **e** Transwell assays were performed to detect the effects of PXN or EZH2 on miR-137 mimics-mediated cell migration and invasion. **f** Western blot assays were conducted to measure the level of EMT-related proteins in indicated cells. **g** Clonogenic survival assay was performed to testify the effects of PXN or EZH2 on miR-137 mimics-mediated radiosensitivity. **h** Flow cytometry analysis was performed to evaluate cell apoptosis rate after indicated transfections. *P < 0.05, **P < 0.01

Apart from EZH2, PXN was also found to be a direct target of miR-137. Thus, we intended to prove whether miR-137 regulate ESCC cellular processes via targeting EZH2 and PXN. Before rescue assays, we overexpressed PXN in two ESCC cells (Fig. 5a). The expression of PXN or EZH2 was depleted by miR-137 mimics was partially restored by co-transfection of PXN or EZH2 (Fig. 5b, c).

Next, EdU assay indicated that PXN or EZH2 overexpression could recover the proliferative ability weakened by miR-137 upregulation (Fig. 5d). Furthermore, the migratory and invasive capacities impaired by miR-137 upregulation were stimulated again by PXN or EZH2 overexpression (Fig. 5e). The level of EMT-related proteins affected by miR-137 overexpression was reset by the overexpression of PXN or EZH2 (Fig. 5f). Moreover, the surviving fraction reduced by miR-137 mimics was notably increased by PXN or EZH2 overexpression (Fig. 5g). Flow cytometry showed that the radio-induced apoptotic cells were increased by miR-137 mimics, while distinctively decreased again by overexpressing PXN or EZH2 (Fig. 5h). Therefore, we confirmed that CTCF/Suz12/EZH2 complex-silenced miR-137 facilitates ESCC progression and radioresistance by targeting EZH2 and PXN.

## Discussion

MiRNAs play pivotal roles in a wide array of biological processes, such as proliferation, apoptosis and migration [32]. Accumulating evidence supported the involvement of miR-137 in cancer biology [33]. MiR-137 has been found to interact with long non-coding RNA (lncRNA) small nucleolar RNA host gene 1 (SNHG1) in the tumorigenesis of colorectal cancer (CRC) [34]. Moreover, miR-137 has been reported to be abnormally expressed in cancer samples and exhibited anti-cancer potential in ovarian cancer [35] and gastric cancer [36]. Herein, we noticed that miR-137 was notably downregulated in ESCC cells and its upregulation could inhibit cell proliferation migration and radioresistance.

As an 11-zinc-finger nuclear protein, CTCF is a type of insulator protein capable of mediating transcriptional domains through promoter activation or repression [37–39]. In the current study, CTCF was identified to be a transcriptional repressor that was responsible for the downregulation of miR-137 in ESCC. CTCF has been reported to interact with Suz12 and recruit PRC2 complex to transcriptionally suppressing their downstream targets [40]. Polycomb group (PcG) is protein capable of mediating the transcription repressor of target mRNA via chromatin modification. PRC2 is a major principle protein complex of PcG, which can regulate various biological functions. Since Suz12 is a crucial component of PRC2, we hypothesized that CTCF could recruit PRC2 complex and further affect the expression of miR-137. Through our experiments, we elucidated that CTCF could recruit PRC2 complex to promote the DNA methylation in the promoter of miR-137 via the interaction with Suz12.

By means of bioinformatics analysis and molecular mechanical experiments, we identified EZH2 and PXN as two targets of miR-137. Functionally, EZH2 and PXN restoration could counteract the cellular processes and radioresistance mediated by miR-137 upregulation. In conclusion, miR-137 exerts tumor-suppressive functions in ESCC through forming a negative feedback loop with EZH2 and PXN. Our findings may contribute to providing a promising therapeutic target for ESCC patients. We will make clinical investigation on this feedback loop in our future study.

## Conclusion

Our findings revealed the downregulation of miR-137 in ESCC and its tumor suppressive role by targeting EZH2 and PXN. Our findings might provide a promising target for ESCC treatment, especially for patients with radioresistance.

## Supplementary Information

**Additional file 1:**All untrimmed western blots images.

## Data Availability

Research data are not shared.

## Abbreviations

ESCC: Esophageal squamous cell carcinoma
miRNAs: MicroRNAs
EC: Esophageal carcinoma
EA: Esophageal adenocarcinoma
EZH2: Enhancer of zeste homolog 2
PRC2: Polycomb repressive complex 2
PXN: Paxillin
Suz12: SUZ12 polycomb repressive complex 2 subunit
H3K4me3: Histone H3 lysine 4 trimethylation
lncRNA: Long non-coding RNA
miRNA: MicroRNA
CRC: Colorectal cancer
PcG: Polycomb group

## References

[1] Domper Arnal MJ, Ferrandez Arenas A, Lanas Arbeloa A (2015). Esophageal cancer: risk factors, screening and endoscopic treatment in Western and Eastern countries. World J Gastroenterol.

[2] Torre LA, Bray F, Siegel RL, Ferlay J, Lortet-Tieulent J, Jemal A (2015). Global cancer statistics, 2012. CA Cancer J Clin.

[3] Hinrichs CS, van Way CW (2002). Esophageal cancer. Curr Surg.

[4] Hesari A, Azizian M, Sheikhi A, Nesaei A, Sanaei S, Mahinparvar N, Derakhshani M, Hedayt P, Ghasemi F, Mirzaei H (2019). Chemopreventive and therapeutic potential of curcumin in esophageal cancer: Current and future status. Int J Cancer.

[5] Gillies RS, Middleton MR, Blesing C (2010). A reply to evidence-based radiation oncology: oesophagus. Radiother Oncol.

[6] Mirzaei H, Yazdi F, Salehi R, Mirzaei HR (2016). SiRNA and epigenetic aberrations in ovarian cancer. J Cancer Res Ther.

[7] Bannon MJ (2015). Identification of long noncoding RNAs dysregulated in the midbrain of human cocaine abusers. J Neurochem.

[8] Naeli P, Pourhanifeh MH, Karimzadeh MR, Shabaninejad Z, Movahedpour A, Tarrahimofrad H, Mirzaei HR, Bafrani HH, Savardashtaki A, Mirzaei H (2020). Circular RNAs and gastrointestinal cancers: epigenetic regulators with a prognostic and therapeutic role. Crit Rev Oncol Hematol.

[9] Pillai RS, Bhattacharyya SN, Filipowicz W (2007). Repression of protein synthesis by miRNAs: how many mechanisms?. Trends Cell Biol.

[10] Mirzaei H (2017). Stroke in women: risk factors and clinical biomarkers. J Cell Biochem.

[11] Sadri Nahand J, Bokharaei-Salim F, Karimzadeh M, Moghoofei M, Karampoor S, Mirzaei HR, Tabibzadeh A, Jafari A, Ghaderi A, Asemi Z (2020). MicroRNAs and exosomes: key players in HIV pathogenesis. HIV Med.

[12] Nahand JS, Mahjoubin-Tehran M, Moghoofei M, Pourhanifeh MH, Mirzaei HR, Asemi Z, Khatami A, Bokharaei-Salim F, Mirzaei H, Hamblin MR (2020). Exosomal miRNAs: novel players in viral infection. Epigenomics.

[13] Lu J, Getz G, Miska EA, Alvarez-Saavedra E, Lamb J, Peck D, Sweet-Cordero A, Ebert BL, Mak RH, Ferrando AA (2005). MicroRNA expression profiles classify human cancers. Nature.

[14] Volinia S, Calin GA, Liu CG, Ambs S, Cimmino A, Petrocca F, Visone R, Iorio M, Roldo C, Ferracin M (2006). A microRNA expression signature of human solid tumors defines cancer gene targets. Proc Natl Acad Sci USA.

[15] Niemoeller OM, Niyazi M, Corradini S, Zehentmayr F, Li M, Lauber K, Belka C (2011). MicroRNA expression profiles in human cancer cells after ionizing radiation. Radiat Oncol.

[16] Weidhaas JB, Babar I, Nallur SM, Trang P, Roush S, Boehm M, Gillespie E, Slack FJ (2007). MicroRNAs as potential agents to alter resistance to cytotoxic anticancer therapy. Can Res.

[17] Lin X, Wang Y (2018). Re-expression of microRNA-4319 inhibits growth of prostate cancer via Her-2 suppression. Clin Transl Oncol.

[18] Wang Y, Cheng Q, Liu J, Dong M (2016). Leukemia stem cell-released microvesicles promote the survival and migration of myeloid leukemia cells and these effects can be inhibited by MicroRNA34a Overexpression. Stem Cells Int.

[19] Nahand JS, Taghizadeh-Boroujeni S, Karimzadeh M, Borran S, Pourhanifeh MH, Moghoofei M, Bokharaei-Salim F, Karampoor S, Jafari A, Asemi Z (2019). microRNAs: new prognostic, diagnostic, and therapeutic biomarkers in cervical cancer. J Cell Physiol.

[20] Aghdam AM, Amiri A, Salarinia R, Masoudifar A, Ghasemi F, Mirzaei H (2019). MicroRNAs as diagnostic, prognostic, and therapeutic biomarkers in prostate cancer. Crit Rev Eukaryot Gene Expr.

[21] Pourhanifeh MH, Mahjoubin-Tehran M, Shafiee A, Hajighadimi S, Moradizarmehri S, Mirzaei H (2020). MicroRNAs and exosomes: small molecules with big actions in multiple myeloma pathogenesis. IUBMB Life.

[22] Savardashtaki A, Shabaninejad Z, Movahedpour A, Sahebnasagh R, Mirzaei H, Hamblin MR (2019). miRNAs derived from cancer-associated fibroblasts in colorectal cancer. Epigenomics.

[23] Jamali L, Tofigh R, Tutunchi S, Panahi G, Borhani F, Akhavan S, Nourmohammadi P, Ghaderian SMH, Rasouli M, Mirzaei H (2018). Circulating microRNAs as diagnostic and therapeutic biomarkers in gastric and esophageal cancers. J Cell Physiol.

[24] Ding F, Zhang S, Gao S, Shang J, Li Y, Cui N, Zhao Q (2018). MiR-137 functions as a tumor suppressor in pancreatic cancer by targeting MRGBP. J Cell Biochem.

[25] Zhang W, Chen JH, Shan T, Aguilera-Barrantes I, Wang LS, Huang TH, Rader JS (2018). miR-137 is a tumor suppressor in endometrial cancer and is repressed by DNA hypermethylation. Lab Invest.

[26] Zhang W, Chen JH, Shan T, Aguilera-Barrantes I, Wang LS, Huang TH, Rader JS, Sheng X, Huang YW (2018). miR-137 is a tumor suppressor in endometrial cancer and is repressed by DNA hypermethylation. Lab Invest.

[27] Alzrigat M, Jernberg-Wiklund H, Licht JD (2018). Targeting EZH2 in Multiple Myeloma-Multifaceted Anti-Tumor Activity. Epigenomes.

[28] Kang Z, Jifu E, Guo K, Ma X, Zhang Y, Yu E (2019). Knockdown of long non-coding RNA TINCR decreases radioresistance in colorectal cancer cells. Pathol Res Pract.

[29] Liu Q, Wang J, Tang M, Chen L, Qi X, Li J, Yu J, Qiu H, Wang Y (2018). The overexpression of PXN promotes tumor progression and leads to radioresistance in cervical cancer. Future Oncol.

[30] Yan H, Tang G, Wang H, Hao L, He T, Sun X, Ting AH, Deng A, Sun S (2016). DNA methylation reactivates GAD1 expression in cancer by preventing CTCF-mediated polycomb repressive complex 2 recruitment. Oncogene.

[31] Ha M, Ng DW, Li WH, Chen ZJ (2011). Coordinated histone modifications are associated with gene expression variation within and between species. Genome Res.

[32] Li SQ, Wang ZH, Mi XG, Liu L, Tan Y (2015). MiR-199a/b-3p suppresses migration and invasion of breast cancer cells by downregulating PAK4/MEK/ERK signaling pathway. IUBMB Life.

[33] Miao H, Wang N, Shi LX, Wang Z, Song WB (2019). Overexpression of mircoRNA-137 inhibits cervical cancer cell invasion, migration and epithelial-mesenchymal transition by suppressing the TGF-beta/smad pathway via binding to GREM1. Cancer Cell Int.

[34] Fu Y, Yin Y, Peng S, Yang G, Yu Y, Guo C, Qin Y, Zhang X, Xu W, Qin Y (2019). Small nucleolar RNA host gene 1 promotes development and progression of colorectal cancer through negative regulation of miR-137. Mol Carcinog.

[35] Dong P, Xiong Y, Watari H, Hanley SJ, Konno Y, Ihira K, Yamada T, Kudo M, Yue J, Sakuragi N (2016). MiR-137 and miR-34a directly target Snail and inhibit EMT, invasion and sphere-forming ability of ovarian cancer cells. J Exp Clin Cancer Res.

[36] Cheng Y, Li Y, Liu D, Zhang R, Zhang J (2014). miR-137 effects on gastric carcinogenesis are mediated by targeting Cox-2-activated PI3K/AKT signaling pathway. FEBS Lett.

[37] Washington SD, Musarrat F, Ertel MK, Backes GL, Neumann DM (2018). CTCF binding sites in the herpes simplex virus 1 genome display site-specific CTCF occupation, protein recruitment, and insulator function. J Virol.

[38] Labrador M, Corces VG (2002). Setting the boundaries of chromatin domains and nuclear organization. Cell.

[39] West AG, Huang S, Gaszner M, Litt MD, Felsenfeld G (2004). Recruitment of histone modifications by USF proteins at a vertebrate barrier element. Mol Cell.

[40] Li T, Hu JF, Qiu X, Ling J, Chen H, Wang S, Hou A, Vu TH, Hoffman AR (2008). CTCF regulates allelic expression of Igf2 by orchestrating a promoter-polycomb repressive complex 2 intrachromosomal loop. Mol Cell Biol.

