# Supplementary figures and images for: CTCF-silenced miR-137 contributes to EMT and radioresistance in esophageal squamous cell carcinoma
Source: Cancer Cell Int. 2021 Mar 8;21:155. doi: 10.1186/s12935-020-01740-8 (PMC7938596; doi:10.1186/s12935-020-01740-8)

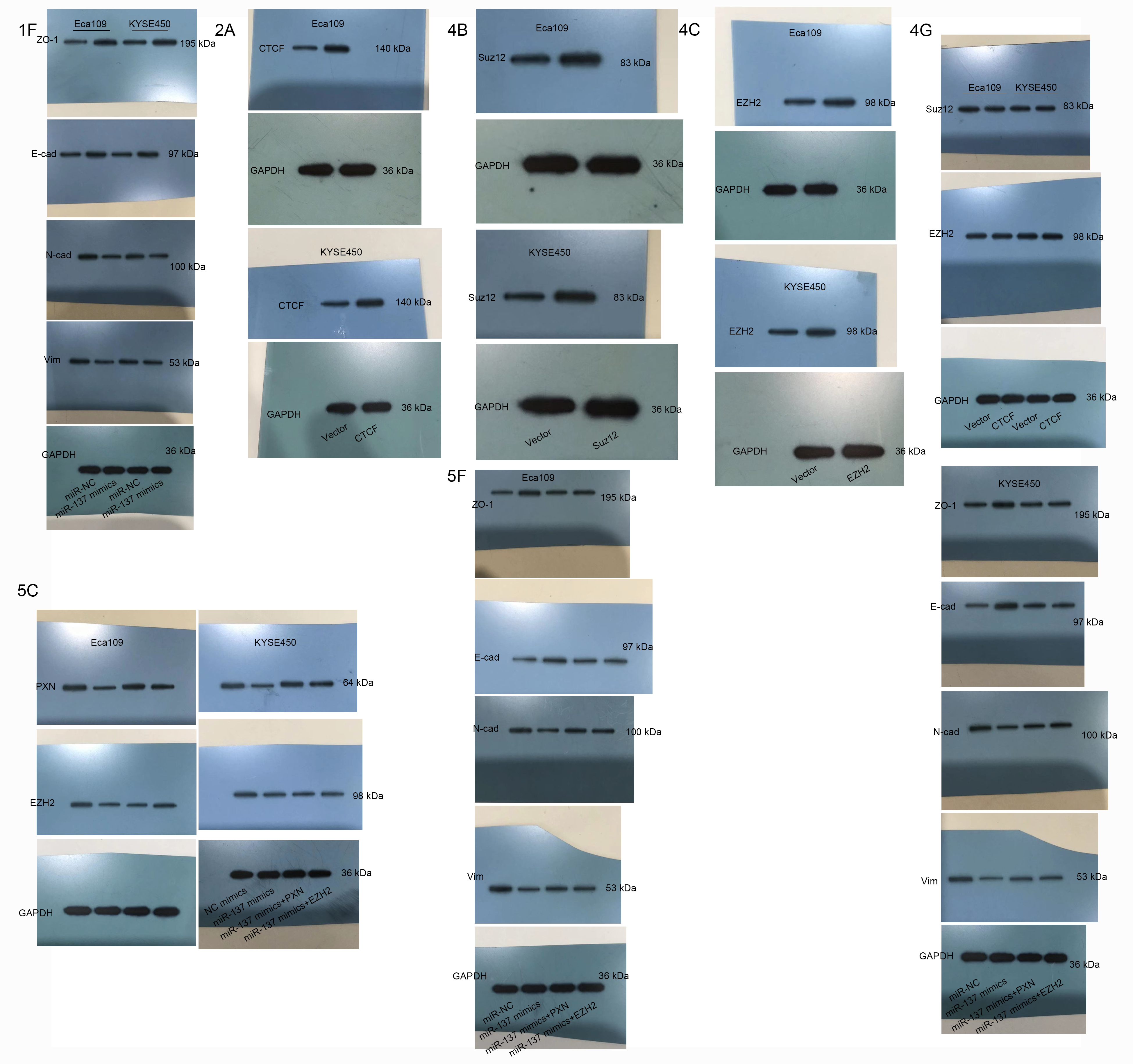

Supplement: Supplementary file 1 — Additional file 1:All untrimmed western blots images. [file 12935_2020_1740_MOESM1_ESM.tif]
